# Serum Inflamma-miR Signature: A Biomarker of Myelodysplastic Syndrome?

**DOI:** 10.3389/fonc.2020.595838

**Published:** 2020-11-20

**Authors:** Marianna Mariani, Domenico Mattiucci, Elisa Rossi, Valeria Mari, Erico Masala, Angelica Giuliani, Valeria Santini, Fabiola Olivieri, Elena Marinelli Busilacchi, Stefania Mancini, Attilio Olivieri, Antonella Poloni

**Affiliations:** ^1^ Hematology Clinic, Department of Clinical and Molecular Sciences, DISCLIMO, AOU Ospedali Riuniti-Università Politecnica delle Marche, Ancona, Italy; ^2^ MDS Unit, Azienda Ospedaliero Universitaria Careggi, University of Florence, Florence, Italy; ^3^ Department of Clinical and Molecular Sciences, DISCLIMO, Università Politecnica delle Marche, Ancona, Italy; ^4^ Center of Clinical Pathology and Innovative Therapy, IRCCS INRCA National Institute, Ancona, Italy

**Keywords:** myelodysplastic syndromes, inflammaging, microRNAs, biomarkers, inflammation

## Introduction

Myelodysplastic syndromes (MDS) are a heterogeneous group of clonal stem cell disorders characterized by ineffective hemopoiesis, peripheral blood cytopenias and a heightened risk (ca. 40% of cases) of developing acute myeloid leukemia (AML) (1). MDS are the most common hematologic malignancies of the elderly, with an incidence over age 80 greater than 50 new cases per 100,000 persons a year and a median survival time from diagnosis of about 30 months (2).

MDS development is a multistep process characterized by cytogenetic changes, gene mutations, or both (3). The mesenchymal niche is emerging as a critical contributor to disease initiation and progression, and disruption of niche cell inflammatory signaling has been suggested to promote cell mutations and selection and to facilitate clonal expansion (4).

Inflamm-aging—a term coined by Claudio Franceschi’s team 20 years ago—indicates the low-grade, chronic, systemic inflammation that characterizes aging in the absence of overt infection, and is recognized as the main risk factor for morbidity in the elderly (5). Not surprisingly, recent work has found an important role for it in the pathogenesis of MDS, a disorder typically affecting the elderly (6).

Mounting evidence has been suggesting that epigenetic changes, which in patients with advanced disease induce widespread gene hypermethylation and histone modifications, play a key role in the development of age-related disorders (7–9). In the past few years, non-coding RNAs, including microRNAs (miRNAs), have also emerged as critical factors modulating gene expression. MiRNAs are involved in several cellular processes such as tumor suppression and the self-renewal and differentiation of hematopoietic progenitors. They are shed from or actively released by cells into the circulation and taken up by recipient cells, functioning as highly efficient systemic communication tools.

A number of miRNAs are involved in inflammatory pathway modulation and have thus been defined as “inflamma-miRs” (10, 11). Inflamma-miRs such as miR-146a and miR-21 as well as miRNAs strongly expressed in endothelial cell lineages and hematopoietic progenitors (“angio-miRs”), like miR-126, have been explored in relation to the most common age-related diseases, including type 2 diabetes (12, 13). The presence of inflamma-miRs in plasma and other body fluids as well as in tissue implicates them in the crosstalk between the tissues and organs affected by systemic inflammation (14).

In this study, the levels of these inflamma-miRs were determined in the serum of patients with MDS, to test their prognostic value also in this group of patients.

## Materials and Methods

### Serum Samples

Whole blood was collected from 68 MDS patients and 68 age-matched healthy donors who provided their written informed consent to be included in the study. All procedures were in accordance with the guidelines of the local ethics committee and followed the Declaration of Helsinki guidelines (Prot. no. 210340, 8/7/2010).

Sixty-eight MDS patients, 37 males and 31 females with a median age of 74 years (range, 32–92 years) were enrolled in the study. Grouping according to the International Prognostic Scoring System (IPSS) showed that 46 patients were at lower risk (low or intermediate-1; LR), whereas 16 patients were at higher risk (intermediate-2 or high; HR) and included AML patients with 20-30% myeloid blasts (RAEB-T according to the FAB classification (15). The karyotype of 6 patients could not be assessed. Sixteen patients had comorbidities (diabetes mellitus, cardiovascular disease or cancer), but their miR levels were not significantly different from those of patients without comorbidities (**Table 1**). Samples were collected at diagnosis, prior to any treatment; the MDS risk group was defined at this time according to the IPSS and the revised IPSS (16, 17).

**Table 1:** Clinical and biological features; six karyotypes were not assessable, nine patients had acute myeloid leukemia (AML) with 20%–30% of blasts in bone marrow (RAEB-t in FAB classification).

Characteristisc	N° 68	%
**Age**		
<65 years	9	13
65–74 years	25	37
>74 years	34	50
**Sex**		
M	31	46
F	37	54
**WHO 2016**		
MDS SLD	13	20
MDS MLD	17	25
MDS RS sld	3	4
MDS RS mld	2	3
MDS EB1	9	13
MDS EB2	8	12
MDS with 5q-	7	10
AML	9	13
**IPSS *score*°**		
Low	26	35
Int-1	20	26
Int-2	3	4
High	4	5
**R-IPSS *score*°**		
Very low	9	12
Low	27	36
Int	9	12
High	7	9
Very high	1	1
**Comorbidity**		
Yes	18	26
No	50	74
**Therapy**		
No therapy	14	22
ESAs	20	28
Lenalidomide	11	15
Hypometilating agents	15	24
AlloSCT	2	3
Other	6	8

The control group consisted of 37 men and 31 women with a median age of 71 years (range: 29–92 years). They were considered healthy if they were negative for type 1 or 2 diabetes, liver disease, renal failure, a history of cancer, neurodegenerative disorders, or infectious or autoimmune diseases.

### RNA Extraction From Serum

Total RNA was extracted from 200 μl of serum using Total RNA purification Kit (Norgen Biotek Corp., Thorold, ON, Canada) following the manufacturer´s instructions and stored at −80°C. The synthetic cel-miR-39, a *Caenorhabditis elegans* miRNA, was spiked into serum before RNA extraction. Only samples with cel-miR-39 recovery > 95% were used in subsequent analyses.

### Reverse Transcription PCR and Real Time PCR

RNAs were reverse transcribed with TaqMan Advanced miRNA cDNA Synthesis Kit (Applied Biosystems, Thermo Fisher Scientific, Waltham, MA, USA) following the manufacturer’s recommendations.

Quantitative Real Time PCR was performed with the 7500 Fast System using TaqMan Fast Advanced Master Mix and TaqMan Advanced miRNA Assays (all from Applied Biosystems).

The plasma levels of circulating miRNAs are reported as relative expression (RE) normalized to the mean of spiked-in miR cel-miR-39-3p. The relative expression of each miR was reported as 2−ΔCt.

A total number of 14 inflamma-miRs were analyzed: miR-17, -9, -22, -152, -335, -19b, -20a, -34a, -146a, -181a, -21, -126, -29a, and -155.

### Statistical Analysis

The demographic and clinical data were analyzed descriptively by calculating means and standard deviations. The non-parametric test for two independent samples (Mann-Whitney U test) was used to compare MDS patients with and without comorbidities and to compare miRNA expression between patients (all subtypes) and controls. The correlation between relative miRNA expression and samples (controls and MDS grouped according to IPSS scores) was tested by one-way analysis of variance (ANOVA). The statistical dependence of expression among miRNAs was calculated with Spearman’s rank correlation coefficient (r_s_). Pearson’s parametric correlation was used to relate miRNA expression to patient characteristics.

Overall survival (OS) was measured from the day of diagnosis to the date of the last follow-up or death from any cause. The distribution of OS curves was estimated using the Kaplan–Meier method and groups were compared using the log rank test. Statistical analysis was performed using SPSS version 21.0 (IBM Corp., Armonk, NY, USA). For all analyses, a p value < 0.05 (< 0.01 for Spearman rank correlation analysis) was considered statistically significant.

## Results

### MiRNA Expression in MDS Patients

Of the 14 inflamma-miRs studied (miR-17; -9; -22; -152; -335; -19b; -20a; -34a; -146a; -181a; -21; -126; -29a; -155), only miR-19b, miR-21, miR-146a, miR-155, and miR-181a were detected in patients and controls and showed dysregulation in patients.

Compared to controls, patients showed significantly lower miR-155 (p=0.048) and miR-181a (p=0.049) levels and significantly higher miR-19b (p=0.02) levels.

**Figure 1:**
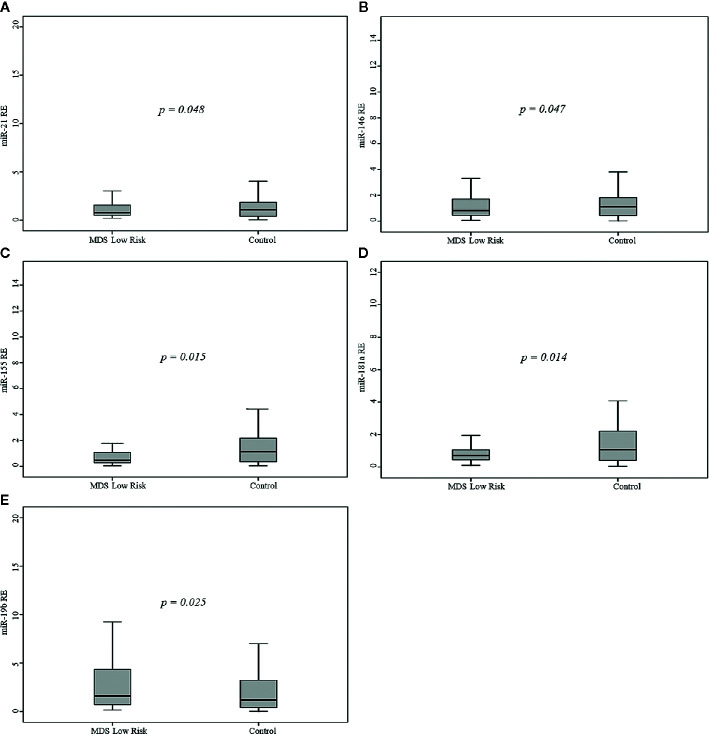
Relative expression (RE) of the five microRNAs (miRNAs) in 68 myelodysplastic syndromes (MDS) patients and 68 healthy controls. **(A)** miR-21, **(B)** miR-146a, **(C)** miR-155, **(D)** miR-181a, **(E)** miR-19b.

Comparison of controls to the MDS risk groups demonstrated that LR patients had significantly lower miR-21 (p=0.048), miR-146a (p=0.047), miR-155 (p=0.015), and miR-181a (p=0.014) levels and significantly higher miR-19b (p=0.025) levels (**Figure 1**), whereas the difference between HR patients (including AML patients) and controls was not significant. LR patients had significantly lower levels of miR-21 (p=0.02) and miR-181a (p=0.049) than AML patients.

The correlation matrix of the five miRs showing a differential regulation is reported in **Table 2**. Notably, significant positive correlations were found between the levels of miR-21 and of the other miRs: miR-146a expression correlated with the levels of miR-21 and miR-181a; miR-155 expression correlated with miR-21 and miR-181a levels; and miR-181a expression correlated with miR-21, miR-146a, and miR-155 levels. MiR-146a expression was related to platelet count and miR-19b expression to hemoglobin levels. The levels of miR-21, miR-155, miR-181a were not related to any other patient characteristic.

**Table 2:** Correlation of microRNA (miRNA) levels.

	miR-21	miR-146a	miR-155	miR-181a	miR-19
**miR-21**					
r_s_	1	0.310	0.516	0.680	0.345
* p* value		***0.013***	***<0.001***	***<0.001***	***0.005***
**miR-146a**					
r_s_	0.310	1	0.230	0.346	0.097
*p* value	***0.013***		0.680	***0.005***	0.448
**miR-155**					
r_s_	0.516	0.230	1	0.589	0.161
*p* value	***<0.001***	0.68		***<0.001***	0.204
**miR-181a**					
r_s_	0.680	0.346	0.589	1	0.104
p value	**<0.001**	***0.005***	***<0.001***		0.413
**miR-19**					
r_s_	0.345	0.097	0.161	0.104	1
p value	**0.005**	0.448	0.204	0.413	

r_s_: Spearman’s correlation coefficient.

The bold and italic values are the statistically significant values.

### Prognostic Significance of miRNA Expression in MDS Patients

**Figure 2:**
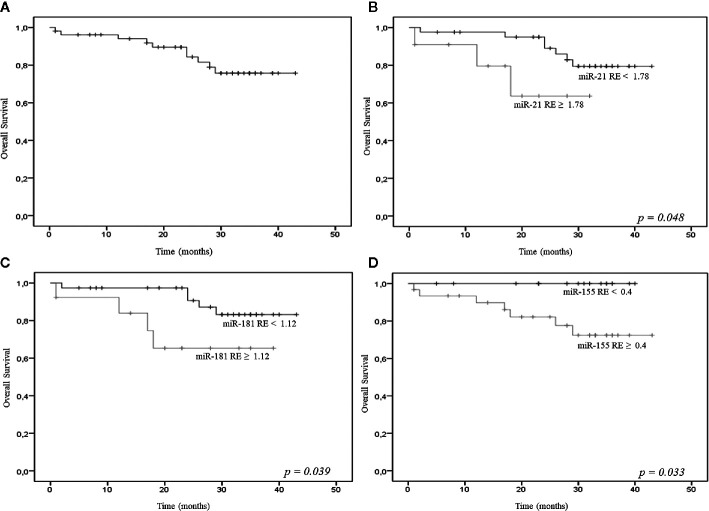
**(A)** Overall survival (OS) of the 68 patients [all myelodysplastic syndromes (MDS) subtypes]; relative expression (RE) levels of miR-21 **(B)**, miR-181a **(C)**, miR-155 **(D)** and OS.

At a median follow-up of 28 months (range, 0 to 43 months), the 2-year OS probability of the 68 patients was 83% (**Figure 2A**); in addition, at a median follow-up of 31 months (range, 1 to 45 months), the 2-year OS probability of the 46 LR patients was 86% (data not shown).

Empirical cut-off values were calculated for miR-21, miR-181a, miR-155, miR-146a, and miR-19b, but only the first three showed significant results. The cut-off values were 1.78 for the 2^delta miR-21, 1.12 for the 2^delta miR-181a, and 0.4 for the 2^delta miR-155. The resulting groups of patients showed significantly different OS probabilities; in particular, the 2-year OS probability was significantly lower for patients with higher miR-21 (62% vs 95%), miR-181a (63% vs 98%), and miR-155 (82% vs 98%) expression (**Figures 2B–D**).

## Discussion

Inflamma-miRs are miRNAs involved in inflammatory pathway modulation in age-related diseases and are highly expressed in hematopoietic progenitor and endothelial cells. Because of their stability in blood, circulating miRNAs are considered as practical and cost-effective candidate biomarkers of the development and progression of the most common age-related disorders (14).

In this study, the serum expression of 14 inflamma-miRs was determined in patients with MDS and in healthy age-matched controls. MiR-21, miR-181a, miR-155, miR-146a, and miR-19b showed a significant differential expression in patients compared to controls.

LR patients, who were the majority (n=46), showed significantly lower serum levels of miR-21, miR-181a, miR-155, and miR-146a and significantly higher levels of miR-19b compared to controls. Notably, miR-21, miR-155, miR-181a levels were not related to any other clinical or biological patient characteristic.

Critically, 2-year OS probability was significantly lower for patients with higher miR-21, miR-181a, and miR-155 expression than for those with lower levels of these miRs. MiR-21 and miR-181a were significantly higher in AML patients than in LR patients.

MiR-21 is an “onco-miR”, and as such has been the subject of extensive research. It regulates cell proliferation, differentiation and apoptosis by modulating target genes including tropomyosin 1 (TPM1), programmed cell death gene 4 (PDCD4), transforming growth factor (TGF-b) pathway genes, Bcl-2, and phosphatase and tensin homolog (PTEN) (18, 19). Measurement of its level in bone marrow from MDS patients suggested that it may stimulate hematopoiesis *in vitro* and *in vivo* and that it may play a critical role in regulating MDS myelosuppressive pathways (20).

MiR-181a is overexpressed in AML patients compared to healthy controls and the whole miR-181 family is overexpressed in high-risk compared to low-risk MDS patients. A signature of 10 miRNAs closely associated to the IPSS risk groups has been able to discriminate between lower- and higher-risk MDS. Selective overexpression of miR-181 family members has been described in higher-risk MDS patients, indicating an overlap with AML pathogenesis. Moreover, lower-risk MDS patients showing a higher expression of miR-181 family members had a significantly lower median survival than patients with lower miR-181 expression (21). The miR-181 family plays an important role in the negative regulation of hematopoiesis, in stem/progenitor cell proliferation and differentiation and in the development of megakaryocytic lineages (22). Cattaneo and co-workers have documented that an initial upregulation of miR-181a-5p and miR-181b-5p during maturation from CD34+ CD38- hematopoietic stem cells to CD34+ CD38+ hematopoietic progenitors is followed by strong downregulation during maturation to granulocytic or monocytic lineages. The miR-181 cluster (miR-181a-3p, -5p and b-3p) showed a varied expression in their AML patients; in particular, it was high in the RUNX1/RUNX1T1-positive AML subtype, whereas only miR-181a-5p showed a steep increase in acute promyelocytic leukemia, where miR-181a-3p was usually downregulated. AML patients with cytogenetic abnormalities also exhibited heterogeneous miR-181a/b levels (23). Marcucci and colleagues reported that the expression of five miR-181 family members was inversely associated with progression-free survival in AML patients with normal cytogenetics (24).

Bone marrow cells (BMCs) were not analyzed in our patients. The different miRNA levels determined in serum (this study) and BMCs in other studies may be explained by the different sample type. However, miR-21 and miR-181a were overexpressed in AML and their high levels predicted a lower OS probability, in line with previous reports. These findings confirm that the expression of these two miRNAs correlates with cell proliferation and apoptosis and suggest a link with the blast population in bone marrow and peripheral blood as well as with disease progression.

Kim et al. have also reported lower miR-21 serum levels in 58 MDS patients compared to 14 controls and showed that among patients treated with hypomethylating agents progression-free survival was significantly longer in those with low serum miR-21 than in those with high serum miR-21 (25). Choi et al. have found a significantly lower BMC expression of several miRs, including miR-124a, miR-155, miR-182, miR-200c, miR-342-5p, and Let-7a, in MDS patients than in healthy volunteers; miR-21, miR-146b-5p, miR-126, and miR-155 levels were significantly higher in higher-risk (intermediate-2 and high) than in lower-risk (low and intermediate-1) patients. These data suggest an association of these miRs with MDS progression and leukemic transformation. The authors also reported that higher miR-126 and miR-155 levels correlated with shorter OS and with the risk of transformation to AML (26).

Marcucci et al. have demonstrated that miR-155 levels are an independent prognostic factor in patients with primary normal-karyotype AML (27). O’Connell et al. reported that miR-155 was significantly upregulated in higher-risk MDS. Moreover, mice transplanted with miR-155-transfected stem cells developed a myeloproliferative disorder with abnormal granulocyte morphology analogous to MDS, suggesting a role for this miR in the higher-risk MDS phenotype (28). MiR-155 levels in extracellular vesicles have been reported to be significantly lower in MDS and significantly higher in AML (29). MiR-155 is induced in hematopoietic stem cells and myeloid progenitor cells during inflammatory responses under the transcriptional control of nuclear factor-kB (NF-kB) and activator protein 1 (AP-1) (30, 31). By preferentially targeting inflammation inhibitors, miR-155 sustains the innate immune response. According to Oliva et al, miR-155 and miR-146a were underexpressed in BMCs from MDS patients with 5q deletion at diagnosis, but lenalidomide treatment raised their levels (32). Starczynowski et al. identified toll-interleukin-1 receptor domain-containing adaptor protein (TIRAP) and tumor necrosis factor receptor-associated factor-6 (TRAF6) as targets of miR-145 and miR-146a, respectively, in a commonly deleted region on chromosome 5. Their concurrent loss resulted in activation of innate immune signaling through elevated expression of their respective mRNA targets, TIRAP and TRAF6 (33). All our LR patients showed significantly lower miR-146a levels than controls, confirming the inappropriate activation of innate immune signaling also in these disorders.

Besides the significant underexpression of miR-21, miR-181a, and miR-155, and miR-146a, our MDS patients (whole sample and LR patients) also had significantly higher miR-19 levels. Zhang et al. found significantly increased miR-19a/b levels in bone marrow mononuclear cells from 113 AML patients than in 42 controls and found that miR-19b upregulation was associated with a poor prognosis and AML recurrence (34).

Although the functions of miRNAs are not fully understood, substantial evidence suggests a role as functional biomarkers of MDS for some of them; in particular, some inflamma-miRs identified in the myelodysplastic bone marrow niche appear to be involved in disease development and progression.

Our findings suggest that specific circulating miRNAs or miRNA signatures could serve as prognostic biomarkers of MDS. Further work in this area is clearly warranted, since it has the potential to provide a more accurate prediction of the outcomes of individual patients.

## Data Availability Statement

The raw data supporting the conclusions of this article will be made available by the authors, without undue reservation.

## Ethics Statements

The studies involving human participants were reviewed and approved by Local Ethics Committee of Azienda Ospedaliero-Universitaria Ospedali Riuniti, Ancona. The patients/participants provided their written informed consent to participate in this study.

## Author Contributions

AP designed the study and wrote the manuscript. DM, ER, VM, AG, SM, and EMB performed the experiments. DM, MM, and ER analyzed the data and wrote the manuscript. AP, EM, VS, and FO provided samples and analyzed clinical data. MM, DM, FO, AG, AO, and AP revised the manuscript. All authors contributed to the article and approved the submitted version.

## Funding

This work has been supported by grants from Associazione Italiana Contro le Leucemie, Linfomi e Mieloma (AIL), Sezione di Ancona-ONLUS.

## Conflict of Interest

The authors declare that the research was conducted in the absence of any commercial or financial relationships that could be construed as a potential conflict of interest.
